# Roles for osteocalcin in brain signalling: implications in cognition- and motor-related disorders

**DOI:** 10.1186/s13041-019-0444-5

**Published:** 2019-03-25

**Authors:** Chang Shan, Arijit Ghosh, Xing-zhi Guo, Shu-min Wang, Yan-fang Hou, Sheng-tian Li, Jian-min Liu

**Affiliations:** 1 Department of Endocrine and Metabolic Diseases, Shanghai Clinical Center for Endocrine and Metabolic Diseases, Rui-jin Hospital, Shanghai Jiao Tong University School of Medicine, Shanghai Institute of Endocrine and Metabolic Diseases, Shanghai, China; 2 Bio-X Institutes, Key Laboratory for the Genetics of Developmental and Neuropsychiatric Disorders (Ministry of Education), Shanghai Key Laboratory of Psychotic Disorders, and Brain Science and Technology Research Center, Shanghai Jiao Tong University, Shanghai, China

**Keywords:** Behavior, Bone, Brain, Cognition, Osteocalcin, Parkinson’s disease

## Abstract

It is now generally accepted that the extra-skeleton functionalities of bone are multifaceted. Its endocrine functions came first to light when it was realized that osteoblasts, the bone forming cells, maintain energy homeostasis by improving glucose metabolism, insulin sensitivity and energy expenditure through osteocalcin, a multipurpose osteokine secreted by osteoblasts. Recently, the emerging knowledge on the functional aspects of this osteokine expanded to properties including adult and maternal regulation of cognitive functions. Therapeutic potential of this osteokine has also been recently reported in experimental Parkinson’s disease models. This review highlights such findings on the functions of osteocalcin in the brain and emphasizes on exploring and analyzing much more in-depth basic and clinical studies.

## Introduction

### The peripheral functions of osteocalcin

One of the recent important discoveries on bone biology is that bone can act as an endocrine organ [1]. It is an organ with a delicate structure that contains various cells of mesenchymal origin, such as the osteoblasts, chondrocytes, bone marrow stromal cells and adipocytes [2]. Among these, osteoblasts are the main type of bone cells that regulate the formation of bones [3], wherein osteocalcin (OCN) acts as a marker for osteoblast activity and bone formation [2]. OCN is a non-collagenous, vitamin K-dependent protein that contains three gamma-carboxyglutamic acid (Gla) motifs, which in the presence of calcium, facilitates mineral deposition and bone remodeling [2, 3]. OCN also acts as a regulator of the activity of osteoclasts and their precursors [3]. However, while studying the role of OCN in bone mineralization, a more striking observation on the role of OCN in fat mass regulation was found, which eventually led to the discovery of OCN in energy metabolism [1]. *Lee et al. (2007)* [4] for the first time highlighted the roles of OCN in glucose metabolism by using OCN knockout (*Ocn*^*−/−*^) mouse models, which showed elevated levels of hyperglycemia and glucose intolerance, decreased β-cell function and insulin secretion, decreased insulin sensitivity and adiponectin expression, and increased fat mass and serum triglyceride level. Subsequent studies have further uncovered the role of OCN in regulating male fertility and exercise adaption, further expanding the functions of OCN in endocrine regulation. Lower circulating testerone levels, decreased testis size and weight, reduced number of spermatocytes and increased germ cell apoptosis were found in *Ocn*^*−/−*^ and osteoblast-specific osteocalcin-deficient *(Ocn*_*osb*_^*−/−*^*)* male mice [5], while 3 month-old *Ocn*^*−/−*^ and *Ocn*_*osb*_^*−/−*^ female mice showed reduced exercise capacity compared with WT mice [6].

Mounting evidence from animal studies has demonstrated that OCN, especially in its metabolic active form, i.e. the uncarboxylated OCN, can act on the pancreas, adipose tissue, male gonads and muscle to stimulate insulin secretion, improve insulin sensitivity, male fertility and muscle power through its peripheral receptor G protein-coupled receptor 6a (*Gprc6a*) [6–9]. Mice lacking *Gprc6a* (*Gprc6a*^*−/−*^) replicate many metabolic and fertile phenotypes of *Ocn*^*−/−*^ mice. For example, mice lacking *Gprc6a* in the β-cell lineage are glucose intolerant due to an impaired ability to produce insulin, the regulation of which occurs both in the prenatal β-cell proliferation and during adulthood [7]. Its depletion in leydig cells or myofibers of mice results in the reduced testes size and weight, epididymis and seminal vesicle weights, sperm counts and circulating testosterone levels [5], and a decreased exercise capacity that is of equal severity as seen in Ocn^*−/−*^ mice [6]. All such evidence indicates the importance of OCN in maintaining the metabolic homeostasis and normal fertility and exercise capacity, providing a basis for its translational potiential in the related metabolic, reproductive and movement disorders.

### Osteocalcin signaling in the brain

In addition to these above-mentioned peripheral effects, OCN also has been found to have unexpected central roles in brain development and cognitive functions [10]. OCN, mainly in its uncarboxylated form, can pass through the blood-brain barrier (BBB) and accumulate in the brainstem, thalamus and hypothalamus, and can bind specifically with neurons in these areas influencing various neurotransmitters synthesis and signaling. In the brainstem, OCN is detected to bind in the dorsal and median raphe nuclei that contain serotonergic neurons, thereby increasing serotonin synthesis, inducing calcium flux and activating action potential (AP) frequency. Besides, both γ-aminobutyric acid (GABA) synthesis and AP frequency of GABAnergic interneurons are decreased after OCN treatment. In the midbrain, OCN binds to neurons in the ventral tegmental area (VTA) and facilitates the synthesis of dopamine. In the hippocampus, OCN binds to a *Gpr158/Ga*_*q*_ complex in the neurons of CA3 region and functions in part by inositol 1,4,5-trisphosphate (IP3) and brain-derived neurotrophic factor (BDNF) [11]. A recent study found that the histone-binding protein *RbAp48*, a molecular determinant of age-related memory loss, could control the beneficial actions of OCN by regulating the expression of *Gpr158* and BDNF in the mouse hippocampus [12]. Upon binding, OCN can signal in these neurons to influence the expression of genes that regulate neurotransmitter synthesis. Ex vivo and in vitro experiments confirmed that OCN could act on neurons to decrease the expression of enzyme involving in GABA biosynthesis glutamate decarboxylase 1 (*Gad1)*, increase tryptophan hydroxylase 2 *(Tph2)* and tyrosine hydroxylase *(Th)* expression in the brainstem and midbrain explants, which are the key enzymes responsible for the synthesis of serotonin, and dopamine as well as norepinephrine, respectively. An animal study again revealed that when OCN was administered through the intracerebroventricular (ICV) route to *Ocn*^*−/−*^ mice, without leakage to peripheral circulation, it normalized *Tph2, Th, Gad1* and *Gad2* expression. These results provide the physiological bases for OCN to act directly on the brain [10].

### The roles of osteocalcin in metabolic diseases

It was obvious that the phenotypes displayed by *Ocn*^*−/−*^ mice were similar to that of clinical T2DM, and the beneficial effects on energy metabolism through exogenous administration of OCN in both cellular and rodent models potentiate its therapeutic possibilities in T2DM and other related metabolic diseases. Daily injections of recombinant OCN at either 3 or 30 ng/g/day could improve the glucose tolerance and insulin sensitivity in mice through increased β-cell mass and insulin secretion [13]. Moreover, ex vivo experiments showed that 1 ng/ml and 3 ng/ml undercarboxylated OCN, but not its carboxylated form, significantly stimulated the expression of cyclin D1 and insulin in the islets as well as the expression of adiponectin, one of the major adipokines that facilitates insulin sensitivity, in adipocytes [4]. A later study by the same team found that OCN intervention not only ameliorated glucose metabolism impairements in *Ocn*^*−/−*^ mice, but also regulated β-cell gene expressions and significantly alleviated the deleterious effect on body mass and glucose metabolism of gold thioglucose-induced hyperphagia and high-fat diet in WT mice [14]. More importantly, OCN could not only regulate β cells function in rodents, but also promote the proliferation and differentiation of β cells and thus enchance insulin sensitivity in cultured human islets at a dose ranging from 1.0–15 ng/ml [15]. Besides the direct regulation on β cells, intraperitoneal administration at 7 μg/kg or oral administration at 10 μg/kg of uncarboxylated OCN could also promote the release of glucagon-like peptide-1 (GLP-1) in intestinal epithelial cells via *Gprc6a*, thus indirectly stimulating insulin secretion [16].

Given the beneficial metabolic effects of OCN found in mice, series of cross-sectional and longitudinal studies have explored its potential associations in humans. A large number of studies demonstrated the negative associations between circulating OCN level and fasting blood glucose level, fasting insulin level, insulin resistance and fat mass, and positive associations with insulin secretion, serum adiponectin level and fat-free mass in normal subjects or in patients with type 1 or type 2 diabetes, gestational diabetes, metabolic syndrome, and polycystic ovarian syndrome [17–21]. These endocrine roles of OCN have provided translational possibilities for its potential uses as a predictor or therapeutic target of some metabolic diseases. For example, a long-term study showed that serum OCN was an independent risk factor for the development of diabetes in patients who were not diagnosed with diabetes at baseline but developed T2DM during a 10-year follow-up [22]. Mounting evidence also suggests the relationship between serum OCN and cardiovascular risk factors. Higher baseline total OCN concentrations were reported to be associated with lower abdominal arotic calcification progression and lower mortality [23], whereas a low level of serum OCN was a significant predictor of increased cardiovascular disease events [24]. Moreover, roles of OCN have been found in the regulation of atherosclerosis (low level associated with increased event of atherosclerosis) [25], anti-tumor activity [26], and brain functions (discussed in the later parts of the article).

## Osteocalcin and cognition

### Role of energy metabolism in cognitive functions

Carrier-mediated glucose uptake with minimal insulin regulation and the fundamental requirement for a glycolytic substrate with minimal intracellular storage make neurons susceptible to glucose toxicity [27]. The brain hippocampus, which is mainly responsible for storage, conversion and orientation of long-term memory, is one of the brain areas with the highest expression of glucose transporter (GLUT) 1 and 4 [28]. Both acute and chronic disturbances in glucose homeostasis can cause transient or permanent neuronal damage and thereby cognitive impairments. As glucose uptake in neurons is insulin-independent, intracellular glucose levels are abnormally elevated during hyperglycemic milieu, a condition in which glucose is oxidized to form free radicals and reactive carbonyls, further activating mitogen-activated protein kinases (MAPK) and thereby altering the cellular phenotypes [27]. These molecular changes finally lead to a series of functional outcomes of diabetes, including nerve conduction deficits [29], pain and allodynia [30], and impaired axonal regeneration [31], which are collectively referred to as glucose neurotoxicity.

Ever since 1854, brain was described as a crucial organ for glucose homeostasis in animal models [32]. Over the past decades, accumulated evidence has demonstrated brain as an insulin-sensitive organ with insulin responses in specific areas including the hypothalamus, fusiform gyrus, prefrontal cortices and the hippocampi [33]. The hippocampi are central to the processing of memory content, especially memories of a declarative nature [34]. Evidence from human studies suggests that intranasal administration of insulin may increase cognitive ability in healthy volunteers [35] and patients with T2DM [36] and Alzheimer’s disease (AD) [37]. Brain insulin resistance is associated with over-weight, peripheral insulin resistance, increased aging processes, maternal metabolic dearrangement and genetic background, which cause not only a reduced capacity to lose weight, weakened feeling of satiety and the exacerbated metabolic diseases such as T2DM [32], but also mediate the possible consequences for cognitive dysfunction and an increased risk of developing AD [37, 38].

T2DM is characterized by insulin resistance and/or relative defects in insulin serection and resulting hyperglycemia. Mounting evidence demonstrated an unequivocal link between T2DM and cognitive decline. T2DM is reported in epidemiological studies to increase a 50–100 % risk of AD and a 100–150 % risk of vascular dementia [39]. In addition, a longinitudial research showed that higher glucose levels in elderly participants were associated with an increased risk of dementia occurring years before the dementia diagnosis [40]. Factors such as endocrine, metabolic and vascular abnormalities contribute to the link between T2DM and cognitive dysfunctions, including ischemic cerebrovascular diseases, glucose neurotoxicity, changes in insulin and amyloid metabolism, increased oxydative stress and inflammatory factors [41]. Chronic hyperglycemia and microvascular diseases cause cognitive dysfunction in both T1DM and T2DM, and both the two disorders are associated with cognitive and motor slowing, decrements of similar magnitude on attention and executive functioning, neuronal growth arrest, increased cortical atrophy and microstructural abnormalities in white matter tracts [42]. Thus, maintaining energy homeostasis is especially important for favoring normal cognitive function and the powerful regulations of OCN on energy metabolism as stated above provide the basis for its indirect regulations on cognition.

### Direct regulation of osteocalcin in cognition

Recent studies have demonstrated putative roles of OCN in the regulation of cognition. To test the central effects of OCN on behaviors and neurotransmitters synthesis, 3 female mice models were generated: *Ocn*^*−/−*^, *Ocn*_*osb*_^*ERT2*^ (a mice model with half of serum OCN levels after tamoxifen treatment), and *Gprc6a*^*−/−*^ mice. The first two mouse models demonstrated significant less locomotor activity, increased anxiety- and depression-like behaviors as compared with controls. These passivity behaviors were considered to be caused by increased GABA biosynthesis in the brain due to the facts that the GABA content in the brain, the expression levels of Ga*d1* and *Gad2* in *Ocn*^*−/−*^ mice and tamoxifen-treated *Ocn*_*osb*_^*ERT2*^ mice were elevated. Other neurotransmitters, such as brain serotonin, norepinephrine and dopamine contents were also decreased in the midbrain, cortex and striatum of the OCN-mutant mice, along with the decreased *Tph2* and *Th* expression. However, all these behaviors, the contents of neurotransmitters, and the expressions of the genes responsible for their synthesis in the brain remained normal in *Gprc6a*^*−/−*^ mice. These findings indicated that independent of its known peripheral receptor *Gprc6a*, OCN impacts directly or through an undetermined second receptor on mice behaviors and neurotransmitters production [10].

As stated above, 10 ng/h OCN delivered *via* ICV infusion could reverse the anxiety- and depression-like behaviors fully, and partially the spatial learning and memory deficit in *Ocn*^*−/−*^ mice. Such a favorable function of OCN on learning and memory can also be exerted postnatally and is critical for fetal brain development [10]. As compared with *Ocn*^*−/−*^ mice or even with *Ocn*^*+/−*^ mice, tamoxifen-treated *Ocn*_*osb*_^*ERT2*^ mice displayed less affected spatial learning and memory capacity, and kept an almost intact corpus callosum and hippocampal dentate gyrus structure. It was found that circulating OCN was detectable 2 days (E14.5) before the expression of this gene in the developing skeleton, suggesting that maternal OCN can reach fetal blood circulation. This hypothesis was proved to be true, that all the OCN found in an embryo before E18.5 comes from maternal origin. Further experiments confirmed that only the maternal uncarboxylated OCN could cross placenta efficiently, enter the fetal blood stream, and prevent fetal neuronal apoptosis in the hippocampus. The beneficial role of maternal OCN on behavior, learning and memory were then established in their adult offspring [10].

In another study, it was described that systemic exposure to young blood counter-balanced the age-related decline in cognitive function in old mice [43]. However, it was later shown that when the plasma from 3-month-old *Ocn*^*−/−*^ mice or young mice plasma with depletion of OCN by its antibody was injected into 16-month-old wild-type mice, no improvement of cognitive performance was observed. Instead, the plasma from 3-month-old wild-type mice or plasma from young *Ocn*^*−/−*^ mice with the addition of recombinant uncarboxylated OCN, so called “spiked plasma”, could elicit the improvement in cognitive function and alleviate the anxiety-like behaviors in old mice [11]. Moreover, long-term (2 months) peripheral delivery of OCN in old mice also fully recovered the cognitive deficits, possibly through the stimulation of BDNF expression in the hippocampi of old mice, thus providing a direct evidence that OCN is important for maintaining normal cognitive function [11].

Then it comes an immediate question, which receptor mediates this central functions of OCN? It is apparently not Gprc6a, because the passivity of *Ocn*^*−/−*^ mice was not observed in *Gprc6a*^*−/−*^ mice [10]. With the use of 3 criteria as a filter, 1) same as *Gprc6a*, this new receptor should belong to a G protein-coupled receptor; 2) the receptor should be present in the CA3 region of the hippocampus where it had been previously shown to bind with OCN [10]; and 3) the receptor should not be expressed in any cells where *Gprc6a* mediated OCN signaling, a new receptor, *Gpr158*, for OCN was identified [11]. A series of subsequent experiments in *Gpr158*^*−/−*^ mice, in shRNA*-Gpr158* -treated mice, which had more than 60 % decrease in *Gpr158* protein level, and in compound heterozygous mice (*Gpr158*^*+/−*^; *OCN*^*+/−*^) confirmed that it is *Gpr158* which mediates the regulatory effect of OCN on hippocampal-dependent memory and anxiety-like behaviors [11]. It is noteworthy that *Gpr158* is not expressed in serotonergic neurons of the dorsal and medial raphe of the brainstem where OCN binds, suggesting the possibility of the existence of a third receptor for OCN.

The link between the endocrine functions of osteoblasts and their brain functions were further strengthened with the evidence that mice with haplo-insufficient for *Runx2*, a master gene responsible for osteoblast differentiation and main regulator for OCN expression, also had deficiency in spatial learning and hippocampal memory, and increased anxiety-like behaviors [44]. Therefore, these series of work, for the first time suggested the link between bone, or more specifically, the OCN and brain cognitive function.

## The neuroprotective effect of osteocalcin in movement disorders

While the previous work mainly focused on the role of OCN in improving age-related cognitive decline, preventing anxiety and depression [45], its neuroprotective function in neurodegenerative disorders such as Parkinson’s disease (PD) had not been tested. PD is a common neurodegenerative disorder characterized by classical motor and non-motor dysfunction, with progressive loss of dopaminergic neurons in the substantia nigra (SN) and depletion of dopamine in the striatum as cardinal pathological defects [46, 47]. Lots of efforts have been made to treat or prevent the progression of this complex disease [46].

Inspired by the role of OCN in brain development and cognitive functions, we hypothesized in our laboratory that OCN may also have neuroprotective function in PD, based on the following reasons: *1)* mice with decreased serum concentration of OCN display increased anxiety-like behavior, impaired spatial learning and memory [10, 44]; *2)* OCN can bind with the neurons in the midbrain, and thus facilitating the formation of dopamine neurotransmitters [10]; *3)* more importantly, we did find that the OCN level in the cerebral spinal fluid decreases in PD rat model as compared with control rats [48]; *4)* our previous human study revealed that circulating OCN level declines with increasing age [49], while PD is also a geriatric disorder; and *5)* circulating OCN level is in a positive association with measures of executive functioning in humans as evaluated by neuropsychological test battery [50, 51]. It was thus logical to test whether administering uncarboxylated OCN through the central or peripheral route could have any impact on the motor symptoms of PD [48]. To meet this end, we first established a PD rat model induced by 6-Hydroxydopamine (6-OHDA). Results from open-field test (OFT), which can evaluate the locomotor activity of rodents, demonstrated that when OCN was given intraperitoneally, the gradual decrease of movement distance over time in PD rats was diminished, and was markedly increased at fourth week as compared with control PD rats. The rearing dysfunction in PD rats was also improved upon receiving OCN treatment. Similar findings were documented in cylinder test and elevated body swing test (EBST) test, which are used to assess the locomotor asymmetry of rats after unilateral 6-OHDA-induced dopaminergic neuronal loss [48].

Although OCN can pass through the BBB, we were still interested in testing the direct central action of OCN in PD rats by injection OCN and 6-OHDA into the right striatum. After 6 weeks, the movement distance in the 6-OHDA rats was less than that of the sham group, and this deficit was significantly ameliorated by OCN treatment. Immunostaining and western blot analyses confirmed that the loss of TH^+^ neurons in PD rats in the areas of SN and striatum was markedly improved with the treatment of OCN, either peripherally or centrally [48]. These novel findings clearly indicated that OCN could correct motor dysfunction and dopaminergic neuron depletion in PD.

The molecular basis for such neuroprotective effects of OCN derived from the presence of its receptor in the rat brain and its possible interaction with its receptor. OCN is now known to be having two receptors, *Gprc6a* and *Gpr158*, while in the rat brain, the mRNA expression level of *Gpr158* in SN, striatum, hippocampus, cortex, cerebellum, and brainstem was 5–150 times higher than that of *Gprc6a*, suggesting that similar to mice, *Gpr158* is also the central receptor of OCN in rats. Co-immunoprecipitation results further demonstrated that OCN could bind with *Gpr158* in the striatum, while the amount of *Gprc6a* bound with OCN was much lower [48]. Although the functional consequence of the binding of OCN with *Gpr158* in this PD model has not been explored, it was reported that the βγ subunit of the heterotrimeric G proteins interacts with the dopamine transporter (DAT) and modulates brain dopamine homeostasis by promoting dopamine efflux [52]. Since *Gpr158* is a G protein-coupled receptor (GPCR), and it is widely present in the rat/mouse brain, including the specific regions of SN and striatum, thus whether the physical interaction between OCN and *Gpr158* in the striatum would lead to any functional alterations in the DAT and dopamine neurons is worthy of investigation. Establishing a *Gpr158* knockout PD model is extremely useful to confirm whether OCN truly exerts its neuroprotective efficacy through this central receptor in PD.

Astrocytes and microglia are two most common brain-resident cells associated with chronic inflammatory reactions in the brain, participating in the development and progression of neurodegenerative diseases, such as PD [53, 54]. In line with the less dopaminergic neuronal loss in OCN-treated PD rats, the number of astrocytes and microglia in the SN and striatum was dramatically reduced in these rats, together with a partial decrease of TNF-α and IL-1β in the striatum of PD rats [48]. Since overreactive microglia-induced chronic inflammation is essential for the pathogenesis of PD [55, 56], these findings added credence to the notion that suppressing the activation and proliferation of microglia can reverse dopaminergic neuron dysfunction and improve PD symptoms [57].

**Fig. 1:**
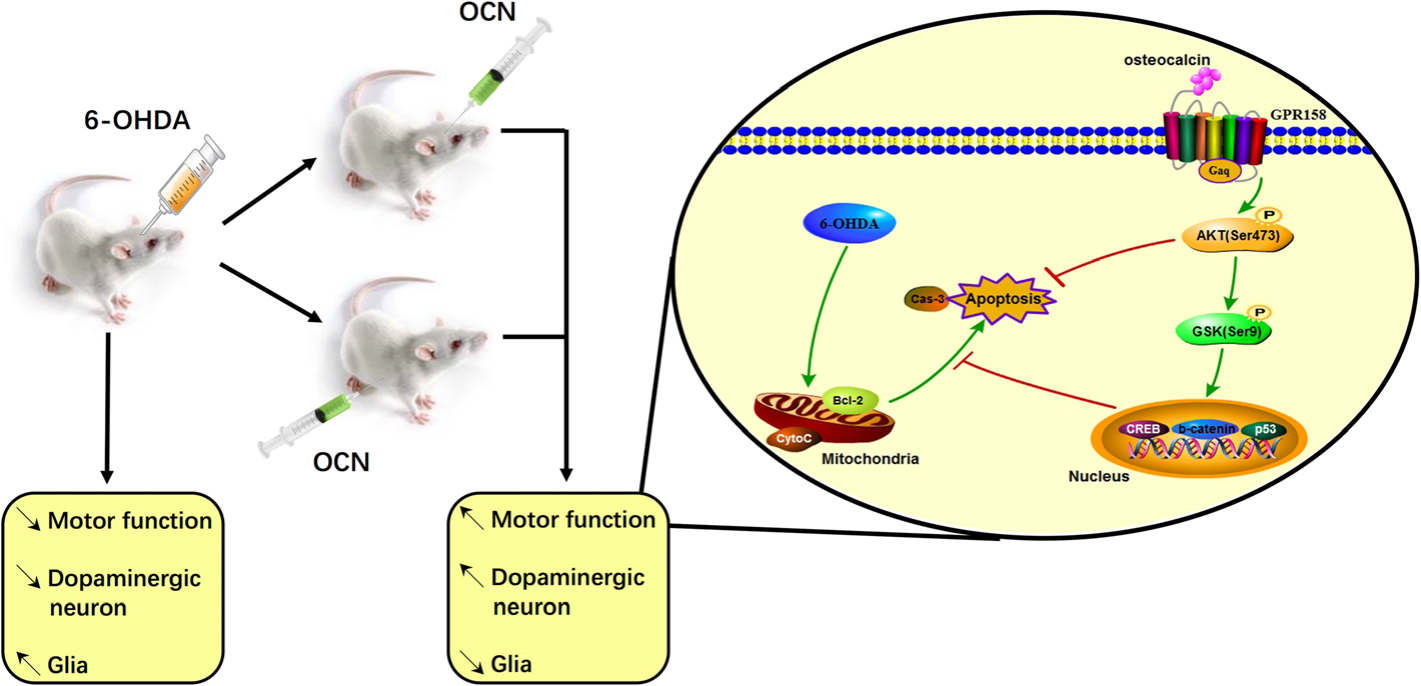
The neuroprotective effects of osteocalcin (OCN) in rats with Parkinson’s disease (PD). OCN, administered either peripherally or centrally, ameliorates motor dysfunction, reduces dopaminergic neuronal loss and diminishes glia-mediated inflammatory responses in PD rat model induced by 6-OHDA *via* the AKT/GSK3β signaling pathway

Finally, in vitro and in vivo data revealed that OCN could reduce dopaminergic neuronal injury induced by 6-OHDA *via* the AKT/GSK3β signaling pathway [48], which was claimed to be one of the pathophysiological mechanisms in the development of PD [58, 59] (Fig. 1).

## Therapeutic implications

### SIRT1 activation by resveratrol on the regulation of osteocalcin and cognition: is there a link?

**Fig. 2:**
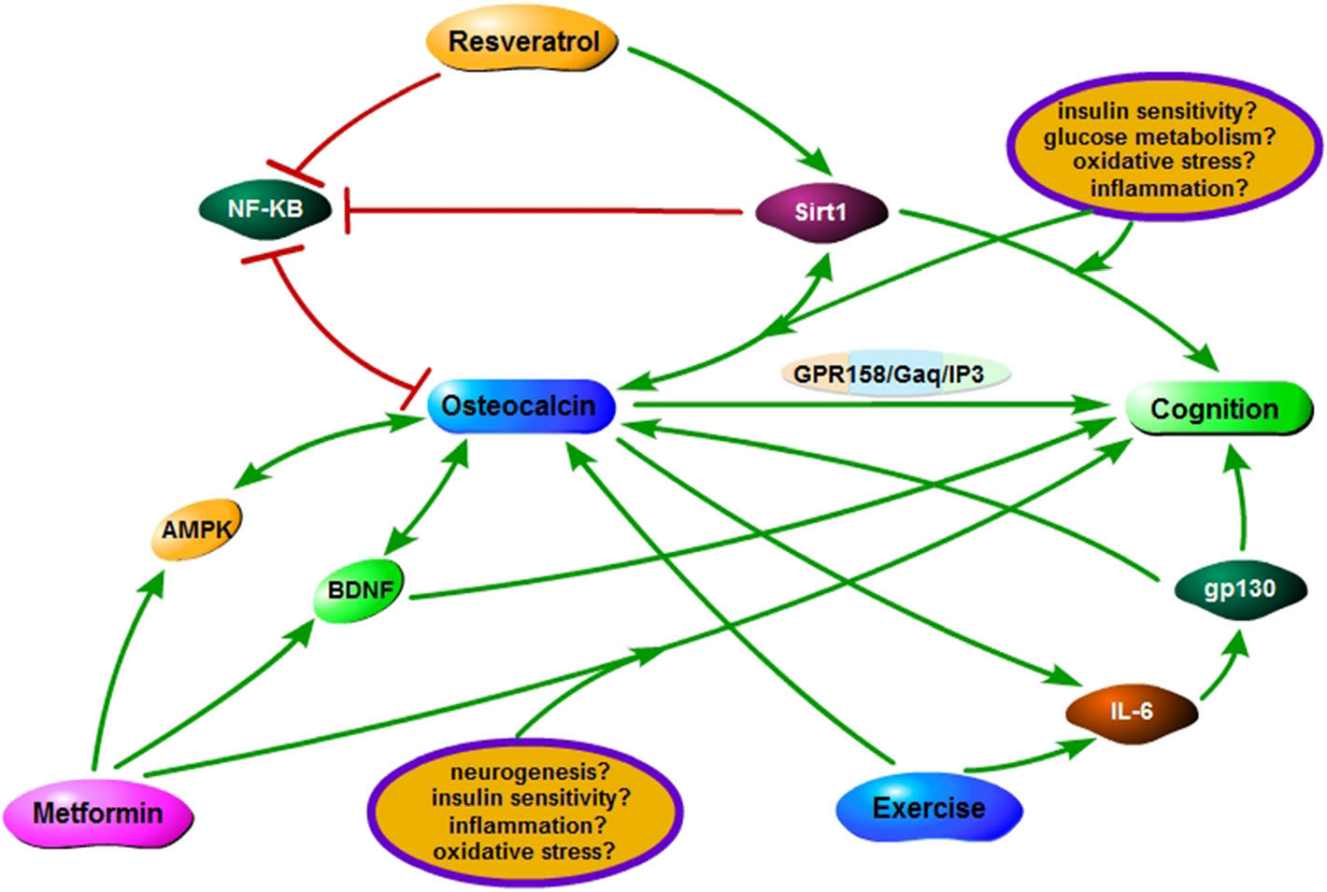
The possible therapeutic implications of resveratrol/metformin/exercise in cognition through osteocalcin signalling. Resveratrol, metformin and exercise have all been reported to have benefical effects on cognition improvements, which might have a link with their regulation on osteocalcin (OCN) from our point of view. Briefly, resveratrol on one hand can induce the expression of OCN, which has been found to improve age-related cognitive decline *via* the GRP158/Gad/IP3 pathway, in human bone marrow mesenchymal stem cells, murine MC3T3 and ST2 cell lines by decreasing nuclear translocation of the NF-κB; on the other hand, several common pathways of SIRT1 activated by resveratrol on the regulation of OCN and cognition, such as improved insulin sensititvity, improved glucose metabolism, reduced oxidative stress and inflammation through the suppression of NF-κB, give rise to the possible link between resveratrol/SIRT1 and OCN. Metformin could attenuate bone loss and increase bone regeneration capability through increased expression of OCN, *via* a mechanism related to AMPK, and regulate behaviours through upregulation of BDNF, which is related to the beneficial actions of OCN in age-related memory loss. These findings suggest a putative link between metformin therapy and brain functions wherein OCN may act as a facilitator of the improved cognitive functions by metformin through improvement of neurotrophic signaling and energy metabolism, and through modulation of inflammatory reactions. An exercise-induced activation of the IL-6/OCN/gp130 axis that in turn improves energy homeostasis and thereby improves cognition could be another plausible explanation of why interventional strategies such as exercise can improve cognitive ability.

SIRT1 is one of the seven sirtuins that act as NAD^+^-dependent histone deacetylase. Resveratrol is a naturally occurring compound, mainly found in red wine obtained from grapes, that mediates its effects on human health through the activation of SIRT1. In the recent years, many studies have confirmed that intake of red wine or direct treatment with isolated resveratrol in humans as well as laboratory animals improve cognitive functions. The compound resveratrol is uptaken by the BBB and is well tolerated in humans [60]. In rat models of vascular dementia, resveratrol has been shown to improve cognition through reduction of oxidative stress [61]. With a 52-week treatment of resveratrol in mild-moderate patients of AD, it was implicated that the treatment could be beneficial for prevention of neurodegenerative disorders through modulation of neuroinflammation [62]. However, a recent meta-analysis revealed that resveratrol might be only useful for elevating mood, but not for improving cognition [63]. This could be possible due to an issue related to the bioavailability of resveratrol, and therefore better derivatives of resveratrol such as pterostilbene, or improved drug delivery tools for the compound might be needed [64, 65]. Nevertheless, the current studies still support the notion that there might be a putative role for this compound on improving cognitive functions, which could be confirmed by long-term prospective studies with resveratrol derivatives with better bioavailability and/or efficacy. Now, the question remains whether the action of resveratrol on cognition has anything to do with OCN or not. SIRT1 activation by resveratrol has been reported be associated with OCN-mediated bone function. In rat calvarial osteoblast-like (ROB) cells, it was found that resveratrol could increase mRNA expression of OCN [66]. In multiple myeloma disease, wherein bone formation is dcreased, resveratrol could induce bone formation through inducement of OCN expression in human bone marrow mesenchymal stem cells and by decreasing nuclear translocation of the NF-κB [67], the transcription factor that is often reported to be inducing the pro-inflammatory cytokines in neuroinflammatory diseases [68]. However, opposing results have also been obtained from other studies [69]. Moreover, there is no direct evidence on whether uncarboxylyted OCN is correlated with the effect of SIRT1. Nevertheless, resveratrol has been found in many studies and has been suggested to be beneficial for bone formation through mechanisms related to OCN [70]. If we summarize the role of SIRT1 on the regulation of OCN and cognition, we can find several common pathways that are in common, *e.g.*, improved insulin sensititvity, improved glucose metabolism, reduced oxidative stress and inflammation through the suppression of NF-κB. Therefore, it is highly possible that SIRT1 may play a crucial role in both bone and cognitive health, which could be complementary to each other, through the improvement of these pathways that involves a common regulatory hormone, *i.e. *OCN (Fig. 2).

### Metformin on cognition and behavioral improvement: is there a role for osteocalcin signaling?

Metformin, which is used primarily as an anti-diabetic therapeutic, has recently been studied largely in many other diseases, including those of in the brain and the bones. Given the role of diabetes in the development of age-related cognitive decline, many anti-diabetic medications, especially metformin, recently have been tested for their efficacy on cognitive improvement [71]. In a longitudinal follow-up study in diabetic patients, long-term use of metformin was found to decrease the risk of developing cognitive decline [72]. Similarly, in the Diabetes Prevention Program (DPP), metformin-treated subjects were found to be at lower risk of developing cognitive malfunction compared to the placebo group. Moreover, the treatment was also found to be cognitively safe, which was, however, found otherwise in other studies [73]. In animal models of cognitive impairments and dementia, metformin has been shown to be fruitful through a number of factors. For example, in a recent study on mice, metformin was shown to alleviate spatial memory loss through an enhanced number of post-mitotic NeuN^+^ neurons (*i.e.* enhanced neurogenesis) [74]. Another study showed that metformin, either alone or in combination with ursolic acid, ameliorated stress-induced changes in behavioral changes, accompanied with insulin sensitivity, inflammatory and oxidative changes in mice [75]. Interestingly, metformin has also been implicated to be beneficial in PD-related mouse model [76]. Insulin sensitivity, which is an important factor in regulation of cognition, can be increased by higher OCN level in clinical diabetic patients [77, 78]. Now the question remains whether there could be a putative link between such beneficial effects of metformin and OCN signaling in cognition. Interestingly, OCN and its related bone diseases have been shown to be affected by metformin. *Liu* et al. *(2018)* found that in a model of ketogenic diet-induced osteoporosis, metformin could attenuate bone loss which correlated with increased level of OCN [79]. The drug has also been shown to improve osteogenic functions of adipose-derived stromal cells, which show increased bone regeneration capability through increasing expression of OCN, *via* a mechanism related to AMPK [80], which is a well known regulator of brain energy metabolism [81] that regulates both cognition and motor coordination [82]. AMPK is known to inhibit NF-κB signaling and inflammation [83], which are related to cognitive and motor impairments, as discussed already before. Moreover, it is of an interesting note that metformin is effective in regulation of behavior through the upregulation of BDNF [76, 84], a brain neurotrophic factor that is related to the beneficial actions of OCN in age-related memory loss [11, 12]. These findings suggest that there might be a putative link between metformin therapy and brain functions wherein OCN may act as a facilitator of the improved cognitive functions by metformin through improvement of neurotrophic signaling and energy metabolism, and through modulation of inflammatory reactions (Fig. 2). However, it should be noted that metformin therapy has also been found to be adverse or ineffective for cognitive functions [85] as well as for the regulation of OCN levels [86]. Therefore, more studies with acute and chronic treatment of the drug in models of cognitive loss and/or its relation with OCN would be needed to understand the role of metformin on OCN/cognition regulatory axis.

### Exercise intervention

A role for OCN in exercise-induced cognitive improvement may be implicated as well. Physical exercise intervention is a well known preventive measure for dementia and aging [87]. Both in young and aged subjects, exercise mediates beneficial effects on cognitive functions regardless of their ages [88]. Using a multi-modal MRI technique, *Den ouden* *et al.* *(2018)* found that aerobic exercise training for 12-week increased left hippocampal volume and facilitated immediate verbal recall performance in humans [89]. Cognition-related disorders, such as AD, are known to be affected by lowered insulin sensitivity and increased inflammatory load in the brain; presence of diseases such as obesity and metabolic syndrome gives rise to systemic inflammation which eventually passes through the BBB. In such scenario, pro-inflammatory cytokines such as TNF-α and IL-1β turn to be dominative while expression of anti-inflammatory cytokines such as IL-4 and IL-10 are decreased [90]. Therefore, shifting the load toward anti-inflammatory dominance from pro-inflammatory dominance may be an useful strategy for improving cognitive function and other brain processes [91]. Now, factors that can improve metabolic status in the brain and muscle tissues may reduce such inflammation. Interestingly, IL-6, which possesses both pro- and anti-inflammatory properties [92], has been found to be related to mediating exercise-induced OCN-mediated energy utilization in muscle [6]. The investigating team on this found that OCN not only could improve the muscle utilization of glucose and fatty acids, which are important factors in controlling cognition and behavior [93], but it could also induce secretion of IL-6 from the muscle fibers [6]. IL-6 is a known regulator of energy and glucose homeostasis that in obese mice increases energy sensitivity through a mechanism related to glycoprotein 130 (*gp130*) [94], which is a regulator of OCN level in osteoblasts [95]. This could be another plausible explanation of why obese animals show low cognitive performance, and interventional strategies such as exercise can improve cognitive ability in obese animals [96]; it might be possible through an exercise-induced activation of the IL-6/gp130/OCN axis that in turn improves energy homeostasis and thereby improves cognition (Fig. 2).

## Future directions

All these results expanded the dimension of bone physiology, and provided with solid evidence to show the novel neuromodulatory functions of OCN in brain development, neurotransmitter synthesis, mood, learning behaviors and locomotor activity. If proved to be true in humans, these findings may have great clinical implications. Elderly people are usually at high risk of developing osteoporosis, cognitive function deficit, sarcopenia, and PD; patients with glucose-related disorders, such as diabetes and metabolic syndrome are also prone to suffer cognitive decline or PD [97–99]; and older adults with motoric cognitive risk syndrome, a recently described predementia syndrome characterized by slow gait with cognitive complaints, has been implicated as a major predictor of cognitive decline and dementia [100], thus, could all these disorders have the common soil, that is, lack of OCN? Or collectively, could we simply name them as “Osteocalcin Syndrome” or “Osteocalcinopathy”? From the treatment perspective, evidence from experimental studies is encouraging, wherein OCN can stimulate insulin secretion, lower glycaemia, improve insulin sensitivity [101], enhance muscle power in old mice [6], ameliorate cognitive dysfunction [11], and is effective in alleviating the motor symptoms of PD [48]. Although there is a long way to go and much more to explore, the potential use of OCN-based therapy in PD and other neurodegenerative disorders is definitely worthy of further investigations in animal models and humans.

## Abbreviations

6-OHDA: 6-Hydroxydopamine
AD: Alzheimer’s disease
AMPK: Adenosine 5′-monophosphate-activated protein kinase
AP: Action potential
BBB: Blood-brain barrier
BDNF: Brain-derived neurotrophic factor
DAT: Dopamine transporter
DM: Diabetes mellitus
EBST: Elevated body swing test
GABA: γ-aminobutyric acid
*Gad*: Glutamate decarboxylase
GLP-1: Glucagon-like peptide-1
GLUT: Glucose transporter
Gpr158: G protein-coupled receptor 158
Gprc6a: G protein-coupled receptor 6a
ICV: Intracerebroventricular
IP3: Inositol 1,4,5-trisphosphate
MAPK: Mitogen-activated protein kinase
OCN: Osteocalcin
Ocn^−/−^: Osteocalcin knock-out
OFT: Open-field test
PD: Parkinson's disease
Runx2: Runt-related transcription factor 2
SN: Substantia nigra
Th: Tyrosine hydroxylase
Tph: Tryptophan hydroxylase
VTA: Ventral tegmental area
